# Local Anaesthetic Infiltration at Trocar Sites for Early Postoperative Pain Control in Laparoscopic Surgery

**DOI:** 10.7759/cureus.101138

**Published:** 2026-01-09

**Authors:** Marta Karczewska, Sara Szukalska, Karolina Lichwala, Angelika Samborska, Barbara Balajewicz, Kamil Wróblewski, Lukasz Siwek, Paulina Wróblewska

**Affiliations:** 1 Orthopaedics and Traumatology, Dr. Tytus Chałubiński Specialist Hospital in Radom, Radom, POL; 2 General Medicine, Medical University of Silesia, Katowice, POL; 3 General Medicine, Poznan University of Medical Sciences, Poznan, POL; 4 Medicine, Municipal Hospital in Zabrze, Zabrze, POL; 5 University Clinical Hospital in Poznan, Poznan University of Medical Sciences, Poznan, POL

**Keywords:** analgesia, bupivacaine, enhanced recovery, laparoscopy, lidocaine, local anaesthesia, postoperative pain, trocar insertion, trocar-site infiltration

## Abstract

Laparoscopic surgery offers clear postoperative advantages compared with open procedures, yet early somatic pain from trocar insertion sites remains a frequent concern. This narrative review examines current evidence on the effectiveness of local anaesthetic infiltration at trocar sites as an early postoperative analgesic strategy. Searches of PubMed, Scopus, and Google Scholar identified studies evaluating pain outcomes during the first 24 postoperative hours across various laparoscopic procedures. Most trials demonstrate that local infiltration provides meaningful early pain reduction, particularly within the first 6-12 hours, regardless of whether anaesthetic is administered before incision or at closure. Longer-acting agents such as bupivacaine, levobupivacaine, and ropivacaine generally yield more sustained relief than lidocaine. Although infiltration does not replace multimodal analgesia, it reliably enhances early comfort, may reduce opioid requirements, and aligns well with enhanced recovery principles. The technique is simple, low-risk, and easily integrated into routine laparoscopic practice, making it a practical adjunct for optimizing immediate postoperative recovery.

## Introduction and background

Over the past 30 years, laparoscopy has gradually replaced open surgery in many abdominal and pelvic procedures. This change did not happen overnight; it followed improvements in instrumentation, growing surgical experience, and perhaps most importantly, a steady accumulation of data showing that patients recover better after minimally invasive operations [1]. For many procedures, laparoscopy has become the default choice. The typical patient experiences less postoperative discomfort, gets out of bed earlier, and often leaves the hospital within a day or two, which aligns with modern expectations of fast-track surgery [2,3].

As techniques have matured, perioperative management has also evolved. The focus today is not only on performing the procedure safely, but also on how the patient feels in the hours and days that follow. Among many aspects influencing recovery, postoperative pain remains one of the most noticeable, for both patient and surgical team [4]. The origin of this pain is not uniform. Part of it stems from traction on the peritoneum and manipulation of abdominal organs. Another component results from pneumoperitoneum, sometimes responsible for characteristic shoulder discomfort. Yet in the early postoperative period, the pain patients most often describe is the sharp, localized tenderness around trocar sites, a somatic consequence of abdominal wall penetration [5,6].

This type of pain matters clinically. It affects the depth of breathing, the willingness to mobilize, and the ability to resume oral intake. In many cases, it also determines how much opioid analgesia is ultimately required [7,8]. Because it arises directly from tissue trauma at the incision point, port-site pain lends itself well to targeted infiltration with local anaesthetic, a technique that aims to interrupt nociceptive signalling at its origin with minimal systemic exposure [9].

Various agents have been studied, lidocaine, bupivacaine, levobupivacaine, and ropivacain, delivered in different concentrations, volumes and at different stages of the operation. A growing interest in pre-emptive infiltration reflects the idea that analgesia may work best when applied before the first incision, limiting the cascade of neural sensitization that follows tissue damage [10,11]. The evidence, however, is mixed. Several randomized trials report lower pain scores and reduced opioid use when trocar sites are infiltrated [12-14], while others find little benefit, particularly in procedures where visceral pain dominates or when short-acting anaesthetics are used [15,16].

Despite variability in outcomes, the method has clear practical appeal. It is inexpensive, requires no specialized equipment, and can be performed easily by the surgeon without prolonging the procedure. Published studies report very few complications, reinforcing its safety profile [17]. In an era of enhanced recovery pathways and increasing pressure to minimize opioid exposure, any low-risk technique that improves comfort, even modestly, deserves a place in discussion. With this in mind, the purpose of this review is to examine what the current body of evidence tells us about trocar-site anaesthetic infiltration and to consider its role in postoperative pain control after laparoscopic surgery.

Materials and methods

Search Strategy

A broad search of the medical literature was conducted in PubMed, Scopus, and Google Scholar, focusing on publications from 2000 up to January 2025, a period during which laparoscopic surgery transitioned from specialist practice to routine clinical standard [1,2]. Search terms included combinations of “trocar-site infiltration”, “port-site anaesthesia”, “local anaesthetic”, “bupivacaine”, “levobupivacaine”, “ropivacaine”, “postoperative pain”, and “laparoscopy”, reflecting terminology commonly used in earlier systematic and clinical trials in this field [1,3,4]. Reference lists of key articles were also examined to identify additional relevant publications, as recommended in narrative evidence synthesis.

Study Selection

Studies were included if they met the following criteria: adult patients undergoing laparoscopic surgery; use of a local anaesthetic infiltrated at one or more trocar sites; postoperative pain assessed with a validated scale such as the visual analogue scale (VAS) or numeric rating scale (NRS) [2,7]; and outcomes reported for at least the first 24 postoperative hours [9,11]. Randomized controlled trials, prospective comparative studies, and relevant reviews were prioritized, as these designs typically offer greater methodological detail and more consistent reporting of pain outcomes [2-4,7]. Titles and abstracts were screened to apply these inclusion and exclusion criteria, with full-text review performed when eligibility could not be determined from the abstract alone. Articles involving paediatric or obstetric populations, robotic procedures, experimental models or interventions unrelated to trocar-site analgesia were excluded, in line with prior reviews addressing postoperative pain pathways in laparoscopy [1,5,8].

Narrative Review Rationale

This review was developed as a narrative synthesis of the available literature on trocar-site local anaesthetic infiltration in laparoscopic surgery. The aim was to identify recurrent clinical patterns, highlight areas of agreement or inconsistency across studies, and outline practical considerations relevant to perioperative pain management. Narrative reviews are particularly suitable in areas where study designs, anaesthetic regimens, and pain assessment methods vary substantially across the literature, making quantitative pooling inappropriate [1,16].

Because this work was conceived as a narrative review, no formal protocol was registered, and no structured assessment of study quality or risk of bias was performed. Instead, emphasis was placed on identifying consistent findings across studies, clarifying areas where evidence diverged, and interpreting results within the broader context of multimodal perioperative analgesia and contemporary enhanced recovery principles [8,12,17]. The heterogeneity of study designs, anaesthetic regimens and outcome measures precluded meta-analysis, and results are therefore presented descriptively.

## Review

Results

Impact on Early Postoperative Pain

Across the studies included in this review, trocar-site local anaesthetic infiltration demonstrated a consistent influence on early postoperative pain, especially within the first postoperative hours when somatic incisional nociception predominates. The majority of randomized and observational trials reported a clear reduction in VAS/NRS pain scores during the first 6-12 hours, particularly in procedures involving multiple trocar ports or larger instrument diameters [2,3,7]. Although the absolute degree of benefit varied from very pronounced analgesic reduction in some cohorts to more modest improvements in others, the overall direction of effect strongly favoured infiltration. These results were most evident when comparing treated patients with controls who received standard systemic analgesia alone [10,11]. As the inflammatory response progresses and the local anaesthetic is metabolized, analgesic impact begins to taper, suggesting that trocar-site infiltration is best conceptualized as an early-phase analgesic intervention rather than a long-lasting solution [12,13].

What makes this finding clinically important is not only the numerical reduction in pain scores but the timing of relief. The immediate postoperative period is often the most difficult for patients, influencing their ability to mobilize, take deep breaths, cough effectively, and participate in early physiologic recovery. Even a moderate decrease in incisional pain during the first several hours can reduce sympathetic activation, minimise respiratory splinting, and improve early ambulation, three outcomes known to correlate with reduced pulmonary complications, faster bowel motility, and earlier discharge readiness [8,9].

The clearest and most consistent analgesic benefit observed across the literature concerns pain experienced shortly after surgery. Several randomized controlled trials demonstrated significantly lower VAS values within two to six hours postoperatively, with some maintaining superiority up to 12 hours [3,9,14]. Relief was most apparent during movement-dependent activities - coughing, standing, turning in bed, getting out of a chair, which aligns well with the somatic origin of trocar-related pain. Importantly, technique mattered. Studies administering anaesthetic pre-incisional demonstrated slightly superior outcomes, consistent with the hypothesis that pre-emptive blockade interferes with nociceptive transmission before central sensitization develops [10,11]. However, this was not universal, as infiltration performed at closure also provided meaningful early benefit. Variability may reflect differences in drug distribution within tissue planes, depth of injection (skin, fascia, peritoneum), and port size, a factor rarely standardized across studies. It is noteworthy that despite technique differences, very few studies showed no benefit at all, highlighting that even imperfectly applied infiltration confers measurable relief when compared with systemic analgesia alone.

Effect on Opioid Consumption and Recovery Metrics

The impact on opioid use was more variable across studies, yet still clinically meaningful. In several trials, patients receiving trocar-site anaesthetic required fewer rescue opioids in the first postoperative night, suggesting that localized pain control can reduce the need for systemic narcotics, even if total 24-hour opioid consumption was not always statistically reduced [7,14]. More importantly, patient-reported comfort and satisfaction tended to improve regardless of whether opioid numbers reached significance. Several investigators also observed faster ambulation, earlier passage of flatus, and quicker tolerance of diet, although these endpoints were reported inconsistently, making meta-level comparison challenging [8,12].

Nevertheless, within the context of enhanced recovery after surgery (ERAS) philosophy, early feeding, ambulation, and minimisation of narcotics, even modest opioid sparing, hold clinical relevance. A reduction of one or two rescue doses may seem small numerically, but it often means avoiding sedation, nausea, delayed recovery, and prolonged hospitalization [17].

Comparison of Anaesthetic Agents

Analgesic duration and depth varied between pharmacologic agents. Lidocaine was effective, but loss of effect often occurred within two to four hours, making it less suited to procedures with prolonged postoperative discomfort [1,4]. Conversely, bupivacaine, levobupivacaine, and ropivacaine maintained relief beyond 6-12 hours, especially when combined with adequate volume and infiltration into deeper fascial planes [9,15]. Studies directly comparing long-acting agents found subtle differences - levobupivacaine and ropivacaine occasionally produced smoother analgesic decline and subjectively better comfort ratings, whereas bupivacaine offered similar intensity but slightly shorter duration [14,16,17]. It is likely that dose, volume, concentration, and depth of deposition matter more than the exact molecule, which may explain why no agent emerged as universally superior.

Comparison with Other Regional Analgesic Techniques

Comparisons with intraperitoneal local anaesthetic instillation revealed mixed outcomes. Some trials favoured trocar infiltration, others found both methods equivalent, while several demonstrated the best analgesia when both techniques were combined - treating somatic and visceral pain simultaneously [8,12]. Stronger contrast appears when comparing infiltration with TAP blocks, QL blocks or epidural analgesia. Regional blocks often provided broader, longer relief, especially when visceral irritation or pneumoperitoneum-related pain was dominant. However, unlike TAP, infiltration is faster, cheaper, technically simple, requires no ultrasound, and can be performed autonomously by the surgeon [10,11]. Practically, infiltration may therefore serve as a default baseline technique, with regional blocks reserved for high-pain procedures or opioid-restricted cohorts.

Safety Profile

Safety findings were universally reassuring. Across all included clinical trials, no cases of systemic local anaesthetic toxicity were documented, and minor effects such as transient burning or numbness resolved quickly without consequence [6,7]. Trials using high-volume infiltration at multiple ports also reported no cardiovascular or neurologic adverse effects, supporting the technique's safety margin even when dosing approaches upper thresholds [4,5]. Given its low risk, rapid execution, and negligible complication burden, trocar-site infiltration is appealing for routine integration into laparoscopic workflows.

**Table 1:** Summary of evidence on trocar-site infiltration VAS: Visual analogue scale.

Study	Technique	Agent	Early Pain Effect	Opioid Use
Cantore et al., 2008 [3]	Pre-incisional	Levobupivacaine	Reduced 6–12h	Lower early need
Altuntaş et al., 2016 [4]	Trocar-site vs intraperitoneal	Bupivacaine	Improved somatic pain	Variable
Çolak et al., 2020 [13]	Preperitoneal infiltration	Bupivacaine	Significant reduction	Reduced
Suragul et al., 2022 [2]	Standard trocar-site infiltration	Local anesthetic (agent not specified)	Lower VAS early	Reduced rescue doses

Discussion

The evidence reviewed in this work suggests that trocar-site infiltration provides a measurable reduction in postoperative pain, most notably during the first postoperative hours after laparoscopic surgery. This pattern was consistent across the majority of randomized and prospective trials, particularly in procedures where pain originates primarily from abdominal wall trauma rather than visceral manipulation [2,3,7]. Because trocar-related pain has a strong somatic component, it responds well to direct blockade of nociceptor pathways at the incision site. This may help explain why infiltration performed before skin incision, functioning as pre-emptive analgesia, appears slightly more effective than infiltration performed at closure. Interrupting afferent signalling prior to tissue injury likely reduces central sensitization, modifying the pain experience during emergence from anaesthesia and the initial recovery window [10,11,14].

**Table 2:** Sources of postoperative pain and the effectiveness of trocar-site infiltration This table is an original synthesis created by the authors based on previously published studies [3,6,9,12].

Source of Pain	Pain Character	Effectiveness of Infiltration
Trocar-induced abdominal wall injury	Somatic pain	Yes, significantly
Peritoneal irritation	Visceral pain	Not significantly
Muscle and fascial stretching	Mixed pain	Partial benefit

One consistent observation across reviewed studies is that this analgesic benefit, while clinically meaningful, is time-limited. Pain scores are typically improved for 6-12 hours after surgery and often return toward baseline within 24 hours [9,12]. This temporal profile reflects both the pharmacokinetics of the drugs used and the evolving nature of postoperative pain: somatic incisional pain dominates early, while visceral distension, pneumoperitoneum-related irritation, and inflammatory components become more relevant later. Accordingly, trocar-site infiltration should be regarded as a short-duration, early-phase analgesic tool, useful for improving comfort immediately after surgery but unlikely to replace systemic analgesia over the full postoperative course [7,13,15].

This also supports its integration into multimodal analgesia strategies, rather than its use as a standalone technique. Combining infiltration with non-steroidal anti-inflammatory drugs (NSAIDs), paracetamol, intraperitoneal anaesthetic instillation, or regional blocks has been proposed in multiple trials, some of which demonstrated additive or synergistic reductions in pain and opioid use [8,12]. Such combinations align closely with ERAS principles, where minimizing opioid exposure, encouraging early feeding and mobilization, and reducing physiological stress are central goals. Even modest early pain reduction can help patients sit up, breathe deeply, and ambulate sooner, factors strongly associated with reduced pulmonary complications and shorter hospital stay.

The variability among published results deserves specific discussion. Differences in drug type, concentration, injection depth, port size, volume administered, and timing likely account for the heterogeneity observed. Lidocaine, while widely available and familiar, provides analgesia lasting only a few hours [1,4]. In contrast, bupivacaine, levobupivacaine, and ropivacaine produce more prolonged relief, and several trials describe incremental advantages in comfort duration when these longer-acting formulations are used [9,15,16]. However, none emerged as definitively superior across studies, indicating that technique, including fascial vs subcutaneous infiltration, pre-incisional vs post-closure timing, and distribution across multiple ports, may influence outcomes as strongly as drug choice itself.

In comparison with regional techniques such as the transversus abdominis plane (TAP) block, erector spinae plane (ESP) block, or spinal/epidural anaesthesia, trocar infiltration is generally less potent, particularly for procedures where visceral pain predominates [8,10]. However, its advantages lie elsewhere: the method is fast, inexpensive, does not require ultrasound guidance or anaesthetic expertise, and can be performed by the surgeon without prolonging operative time [11]. In outpatient or resource-limited settings, this simplicity becomes strategically valuable. It also avoids risks associated with deeper regional blocks, hematoma, nerve injury, failed block, and hypotension, making it an attractive baseline intervention in routine laparoscopic practice.

Safety outcomes reinforce this position. Across reviewed studies, complication rates were exceptionally low. No episodes of systemic toxicity were documented, even in trials using multi-port infiltration with long-acting anaesthetics [6,7]. Mild adverse reactions, when reported, were self-limiting and clinically insignificant. This profile supports the feasibility of incorporating trocar infiltration into standard laparoscopic protocols without additional monitoring or staff training and suggests that repeated or multi-site administration is unlikely to pose meaningful risk [4,5].

Nevertheless, the current evidence base remains heterogeneous and fragmented. Many published trials involve small sample sizes, limited follow-up, non-uniform pain scoring intervals, and inconsistent reporting of opioid consumption or patient-centred outcomes such as readiness for discharge, return to function, or overall satisfaction. Future research would benefit from standardized analgesic protocols, direct comparison of pre- versus post-incisional dosing, and trials designed to distinguish somatic from visceral pain components. Comparative studies evaluating combined infiltration plus regional block approaches may also clarify whether dual-mechanism analgesia yields superior recovery profiles and facilitates opioid-free perioperative pathways [12,17].

In summary, trocar-site infiltration is not a perfect solution, but it is a dependable one. Its analgesic effect is modest yet clinically relevant, concentrated in the early postoperative phase when comfort matters most. It is inexpensive, reproducible, low-risk, and widely applicable, making it a practical addition to multimodal pain management in laparoscopic surgery. When implemented purposefully, ideally pre-incisional and supported by adjunctive systemic or regional analgesia, trocar-site infiltration can meaningfully improve the patient’s immediate recovery experience without adding complexity or cost.

## Conclusions

Trocar-site infiltration offers a straightforward method to lessen early postoperative discomfort after laparoscopic procedures. Most studies indicate that it provides meaningful reductions in somatic pain during the initial recovery period, particularly when longer-acting local anaesthetics are used. Although the magnitude of benefit varies among techniques and agents, the overall direction of evidence supports its use as part of perioperative pain management. The simplicity and safety profile of this approach make it easy to incorporate into routine surgical practice. While it cannot replace comprehensive multimodal strategies, it can enhance patient comfort in the hours immediately after surgery. These findings suggest that trocar-site infiltration remains a practical adjunct for improving early recovery following laparoscopy.
